# A Medico-Legal Treatise on Malpractice and Medical Evidence: Comprising the Elements of Medical Jurisprudence

**Published:** 1860-05

**Authors:** 


*A Medico-Legal Treatise on Malpractice and Medical Evidence; comprising the Elements of Medical Jurisprudence.* By JOHN J. ELWELL, M.D., Member of the Cleveland Bar. New York: John S. Voorhies. Cleveland, O.: Alfred Elwell & Co. 1860.

Some systematic treatise, properly exhibiting and discussing the rights and liabilities of physicians and surgeons, growing out of the practice of their profession, as well as the important subject of medical evidence, would meet a generally acknowledged want. The author of this work, by reason of his double experience in the professions of law and medicine, is presumed to be peculiarly qualified to supply this want; and having given to the subject, as he says, much labor and long thought, he leaves it to his readers to say how far he has succeeded in his effort.

After speaking of the general principles of law applicable to medical men, and the difficulties inherent in the practice of medicine and surgery, he discusses the subject of malpractice in cases particularly of a surgical character, such as amputations, fractures, dislocations, incised wounds, &c., &c., giving a digest of Prof. Hamilton's reports of cases of deformity after fracture, together with a series of adjudicated cases upon these several points.

The legal responsibilities of druggists in the exercise of their vocation, together with leading cases bearing on these questions, are also set forth.

The subject of malpractice in its criminal aspect is next discussed, and to this several chapters are devoted, embracing detailed cases and decisions of courts, both American and English. A chapter on abortion and fœticide closes the first division of the work.

The second treats especially of medical evidence, in which the importance of the subject, its history, and the rights and responsibilities of medical witnesses, are duly dwelt on, and some judicious and practical hints given to medical men, both in regard to preparation for, and manner of, testifying.

The vexed subject of medical evidence in cases of insanity occupies several chapters, and the difficulties that surround it acknowledged to be prominent and troublesome.

Chapters upon medical evidence in cases of poisoning, infanticide, wounds producing death, rape, and one upon the office and duties of coroners, conclude the volume.

We are constrained to say, in regard to this work, so desirable in kind and praiseworthy in design, that we are disappointed in its execution. So far as concerns the collection and embodiment in one volume of many adjudicated cases, scattered through the books, it may be of value to the profession; but as to elucidating the principles involved in them, or as to setting in a newer or clearer light the many medico-legal questions still undecided, we think they will derive from it but little assistance.

There is a want of thoroughness in the examination, and a lack of appreciation of the nature of the subjects discussed in several portions of the work, that one regrets to notice. Particularly is this the case in that portion treating of the subject of insanity, the great defects of which (and the same may be said of other portions) are those which arise from a limited acquaintance with the literature of the subject; a strange misconception as to who are the recognized medical writers and authorities in this department, and from leaving many points undecided, and poised, as it were, between opposing opinions, without the benefit even of the author's own views upon the case.

It might not be equal to the expectations of the author to have his work termed a valuable book of medico-legal quotations, and yet, in fact, it amounts to but little more. L.

